# Glucosylceramide synthase inhibitors prevent replication of SARS-CoV-2 and influenza virus

**DOI:** 10.1016/j.jbc.2021.100470

**Published:** 2021-02-25

**Authors:** Einat B. Vitner, Hagit Achdout, Roy Avraham, Boaz Politi, Lilach Cherry, Hadas Tamir, Yfat Yahalom-Ronen, Nir Paran, Sharon Melamed, Noam Erez, Tomer Israely

**Affiliations:** Departments of Infectious Diseases, Israel Institute for Biological Research, Ness-Ziona, Israel

**Keywords:** glucosylceramide, sphingolipids, antiviral drugs, glucosylceramide synthase, COVID-19, SARS-CoV-2, CC50, cytotoxicity concentration 50%, CPE, cytopathic effect, DENV, dengue virus, DMSO, dimethyl sulfoxide, DNJ, deoxynojirimycin, DsiRNA, Dicer-substrate siRNA, GCS, glucosylceramide synthase, GlcCer, glucosylceramide, GZ-161, GENZ-667161, GZ-346, Genz-123346, LSD, lysosomal storage disease, MDCK, Madin–Darby Canine Kidney, MOI, multiplicity of infection, NB-DNJ, N-butyl–DNJ, PR8, influenza virus A/PR/8/34 (H1N1), SARS-CoV-2, severe acute respiratory syndrome coronavirus 2, SI, selective index

## Abstract

The ongoing COVID-19 pandemic, caused by severe acute respiratory syndrome coronavirus 2 (SARS-CoV-2), is a major threat to global health. Vaccines are ideal solutions to prevent infection, but treatments are also needed for those who have contracted the virus to limit negative outcomes, when vaccines are not applicable. Viruses must cross host cell membranes during their life cycle, creating a dependency on processes involving membrane dynamics. Thus, in this study, we examined whether the synthetic machinery for glycosphingolipids, biologically active components of cell membranes, can serve as a therapeutic target to combat SARS-CoV-2. We examined the antiviral effect of two specific inhibitors of glucosylceramide synthase (GCS): (i) Genz-123346, an analogue of the United States Food and Drug Administration-approved drug Cerdelga and (ii) GENZ-667161, an analogue of venglustat, which is currently under phase III clinical trials. We found that both GCS inhibitors inhibit replication of SARS-CoV-2. Moreover, these inhibitors also disrupt replication of influenza virus A/PR/8/34 (H1N1). Our data imply that synthesis of glycosphingolipids is necessary to support viral life cycles and suggest that GCS inhibitors should be further explored as antiviral therapies.

The recent spread of the novel coronavirus, severe acute respiratory syndrome coronavirus 2 (SARS-CoV-2), has created a worldwide public health emergency. In December 2019, the outbreak of this emerging disease (COVID-19) began in Wuhan, China, and rapidly spread. It was declared as a pandemic by the World Health Organization in March 2020 (https://covid19.who.int/). Antiviral drugs which inhibit the replication of SARS-CoV-2 can be used widely to treat patients after infection. Historically, antiviral drug research has mainly focused on targeting viral components because of the perceived specificity of such an approach (1). Thus, it is not surprising that the first drug to be approved against SARS-CoV-2, remdesivir (also GS-5734), is a direct-acting antiviral that inhibits the viral RNA-dependent RNA polymerase (2). However, because the viral life cycle is dependent on the host, specific host mechanisms can also be explored as antiviral targets. Sphingolipids (SLs) are biologically active components of cell membranes and as such are tightly linked to all processes involving membrane dynamics, making them potential key regulators in the life cycle of obligatory intracellular pathogens such as viruses. Glucosylceramide (GlcCer) is the backbone of more than 300 structurally different glycosphingolipids (GSLs) including gangliosides and sulfatides. Its accumulation leads to Gaucher diseases accompanied by chronic brain inflammation and activation of the antiviral immune response (3). Viral-induced elevation of SL levels was shown to be associated with a number of viruses; elevation of GM2-ganglioside and lactosylceramide was shown upon infection with Zika virus and hepatitis C virus, respectively (4, 5). Human cytomegalovirus induces elevation of ceramide and GM2-ganglioside (6), and dengue virus (DENV) induces elevation of ceramide and sphingomyelin (7). In addition, the influenza virus was shown to induce sphingomyelin and GlcCer elevation (8, 9), and suppression of the biosynthesis of cellular SLs results in the inhibition of the maturation of influenza virus particles *in vitro* (10, 11). Drugs targeting SL metabolizing enzymes are currently in use and constantly being developed for treating lysosomal storage diseases (LSDs) and other disorders in which alteration in SL levels are involved in disease pathology (12, 13, 14). This allows a potential repurposing of these already approved drugs as antivirals. In this study, we examined the antiviral activity of two specific inhibitors of UDP-glucose:ceramide glucosyltransferase (glucosylceramide synthase (GCS)), (EC 2.4.1.80), which catalyze the biosynthesis of GlcCer. These inhibitors block the conversion of ceramide to GlcCer, the first step in the biosynthesis of gangliosides and other GSLs.

The following GCS inhibitors were examined: (i) (1R,2R)-nonanoic acid[2-(2′,3′-dihydro-benzo [1,4] dioxin-6′-yl)-2-hydroxy-1-pyrrolidin-1-ylmethyl-ethyl]-amide-l-tartaric acid salt (Genz-123346), termed hereinafter GZ-346. GZ-346 is an analogue of the United States Food and Drug Administration-approved drug eliglustat (Cerdelga), which is indicated for the long-term treatment of adult patients with Gaucher disease type 1 (15), and (ii) (S)- quinuclidin-3-yl (2-(2-(4-fluorophenyl)thiazol-4-yl)propan-2-yl)carbamate (GENZ-667161), termed hereinafter GZ-161. GZ-161 is a specific inhibitor of GCS that can access the central nervous system and has been demonstrated to effectively reduce GSL synthesis (16, 17, 18). GZ-161 is an analogue of venglustat which is currently under clinical trials for the LSDs; Gaucher disease, Fabry disease, and Tay–Sachs disease, and is in a phase 3 pivotal trial for autosomal-dominant polycystic kidney disease (16, 17, 18).

Thus, the antiviral activity of GCS inhibitors was examined for SARS-CoV-2.

## Results

### GCS inhibitors inhibit SARS-CoV-2 replication

**Figure 1:**
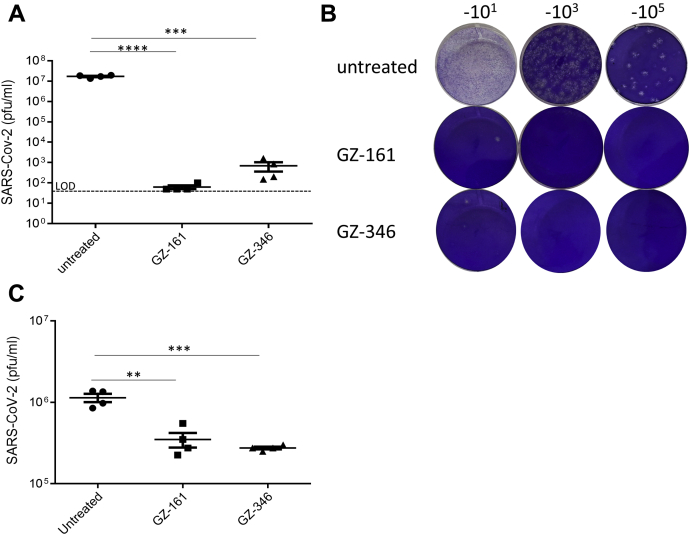
**Inhibition of SARS-CoV-2 by GCS inhibitors.***A* and *B*, Vero E6 cells were treated with GZ-161 or with GZ-346 (10 μM). One hour later, cells were infected with SARS-CoV-2 diluted in Dulbecco's Modified Eagle's medium (MOI, 0.01). Twenty four hours after infection, viral release to the media was measured by the plaque-forming unit (PFU) assay. Data are the means of four replicates ±SEM. ∗∗∗*p* < 0.001, ∗∗∗∗*p* < 0.0001. A representative image of the PFU assay is presented in panel (*B*). *C*, Vero E6 cells were treated with GZ-161 or with GZ-346 (10 μM). One hour later, cells were infected with SARS-CoV-2 diluted in Dulbecco's Modified Eagle's medium (MOI, 5). Ten hours after infection, viral release to the media was measured by the PFU assay. Data are the means of four replicates ± SEM. ∗∗*p* < 0.01, ∗∗∗*p* < 0.001. GCS, glucosylceramide synthase; MOI, multiplicity of infection; SARS-CoV-2, severe acute respiratory syndrome coronavirus 2.

**Figure 2:**
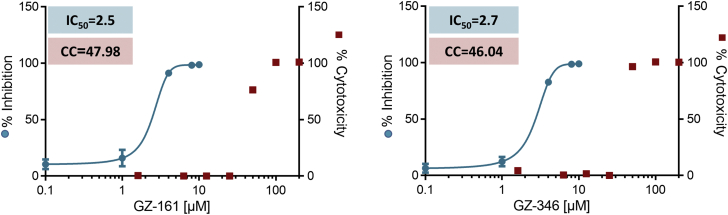
**Dose–response curves of GZ-161 and GZ-346 for inhibition of SARS-CoV-2 infection.** Graphs depict the mean % inhibition of SARS-CoV-2 replication (*blue circles*, *left Y-axis*) and % cytotoxicity (*red squares*, *right Y-axis*) of antivirals. Vero E6 cells were infected in four replicates with SARS-CoV-2 at a multiplicity of infection (MOI) of 0.01 in the presence of drug doses for 24 h, after which viral release was measured through quantitation of SARS-CoV-2 RNA levels by real-time PCR. Cytotoxicity was measured in similarly treated but uninfected cultures *via* the Cell Proliferation Kit (XTT based). Representative data are shown from three independent experiments. The IC_50_ is the concentration on an antiviral required at which virus replication is inhibited by 50% in a cell-based assay. The cytotoxic concentration 50 (CC50) is the concentration of an antiviral agent required to kill 50% of cells in the uninfected culture. SARS-CoV-2, severe acute respiratory syndrome coronavirus 2.

To test for antiviral activity of GCS inhibitors against SARS-CoV-2, Vero E6 cells were incubated with 10-μM GZ-161 or GZ-346 1 h before infection with SARS-CoV-2 (multiplicity of infection [MOI] = 0.01). Supernatants were harvested 24 h after infection and analyzed by plaque-forming units (PFU) assay to measure the effect of the drugs on SARS-CoV-2 replication (Fig. 1, *A* and *B*). Approximately 1.7e7 ± 1.3e6 PFU/ml were detected in the medium of vehicle dimethyl sulfoxide (DMSO) (untreated) infected cells, whereas only 37 ± 23 and 700 ± 339 PFU/ml were detected in GZ-161– and GZ-346–treated cells, respectively, indicating significant inhibition of virus release (*p* < 0.0001, *p* < 0.001, respectively). In addition, the ability of GZ-161 and GZ-346 to inhibit single-cycle infection was studied. Vero E6 cells were infected at a high MOI (MOI = 5), and viral release was determined at 10 h after infection, an early time point of viral release (19, 20). GZ-161 and GZ-346 reduced viral release by 70% and 76%, respectively, even when SARS-CoV-2 were infected with a high MOI (Fig. 1*C*), albeit to a lesser extent than the low MOI. To further determine the antiviral activity and cell cytotoxicity of GZ-161 and GZ-346 against SARS-CoV-2 infection, we measured their IC_50_, cytotoxicity concentration 50% (CC50), and selective index (SI) (Fig. 2). Vero E6 cells were infected with SARS-CoV-2 at an MOI of 0.01 in the presence of serial dilution of GZ-161 or GZ-346. Twenty four hours after infection, the viral copy numbers in the cell culture supernatant were determined by amplifying the nucleocapsid (N) gene by quantitative real-time PCR, and cell viability was measured using the cell proliferation assay (XTT based). The IC_50_ values of GZ-161 (IC_50_ = 2.5 μM) and GZ-346 (IC_50_ = 2.7 μM) were determined at a low micromolar concentration. The SI (CC50/IC_50_) values of GZ-161 and GZ-346 were CC50 = 48 μM, SI > 19.2 and CC50 = 46 μM, SI > 17, respectively (Fig. 2).

**Figure 3:**
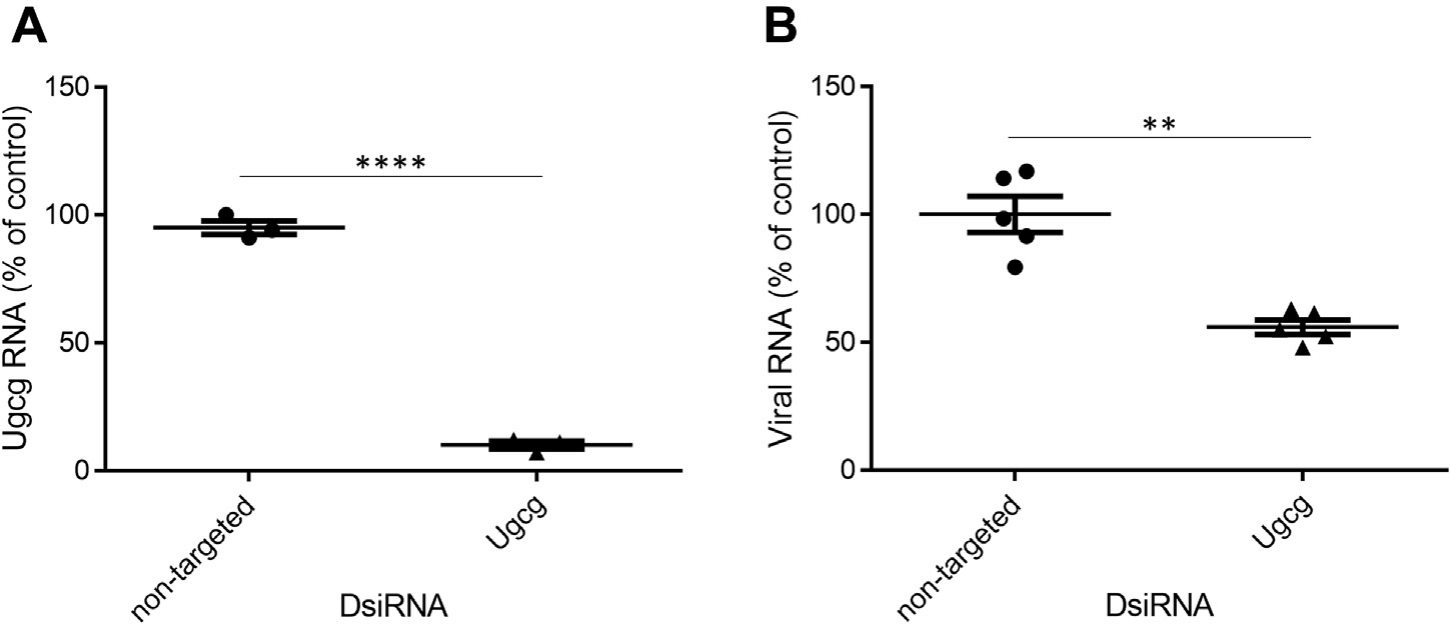
**Knockdown of *Ugcg* gene by DsiRNA reduced SARS-CoV-2 release to the medium.***A*, *Ugcg* silencing efficacy by DsiRNA in Vero E6 cells. Quantification of *Ugcg* mRNAs from nontargeted and *Ugcg*-specific DsiRNA (20 nM) transfected Vero E6 cells 24 h after transfection. mRNA levels were determined by real-time PCR. Mock transfected cells were used as control. Data are the means of triplicates ±SEM. ∗∗∗∗*p* < 0.0001. *B*, 24 h after DsiRNA transfection, cells were infected with SARS-CoV-2 at a multiplicity of infection (MOI) of 0.01. Twenty four hours after infection, viral release was measured through quantitation of SARS-CoV-2 RNA levels by real-time PCR. Data are the means of five replicates ±SEM. ∗∗*p* < 0.01. DsiRNA, Dicer-substrate siRNA; SARS-CoV-2, severe acute respiratory syndrome coronavirus 2.

To demonstrate that inhibition of GCS directly reduces viral replication, *Ugcg* (the gene encoding for GCS) was knocked down by Dicer-substrate siRNA (DsiRNA). The resulting ∼90% reduction in *Ugcg* mRNA expression levels 24 h after transfection (Fig. 3*A*) was sufficient to reduce viral release to the medium by 50% (Fig. 3*B*). The fact that knocking down *Ugcg* inhibits SARS-CoV-2 virus, together with that viral inhibition is observed with two GCS inhibitors with different structures (GZ-161, GZ-346), increases the possibility that the antiviral effect of the two compounds is due to GCS inhibition rather than an off-target effect.

**Figure 4:**
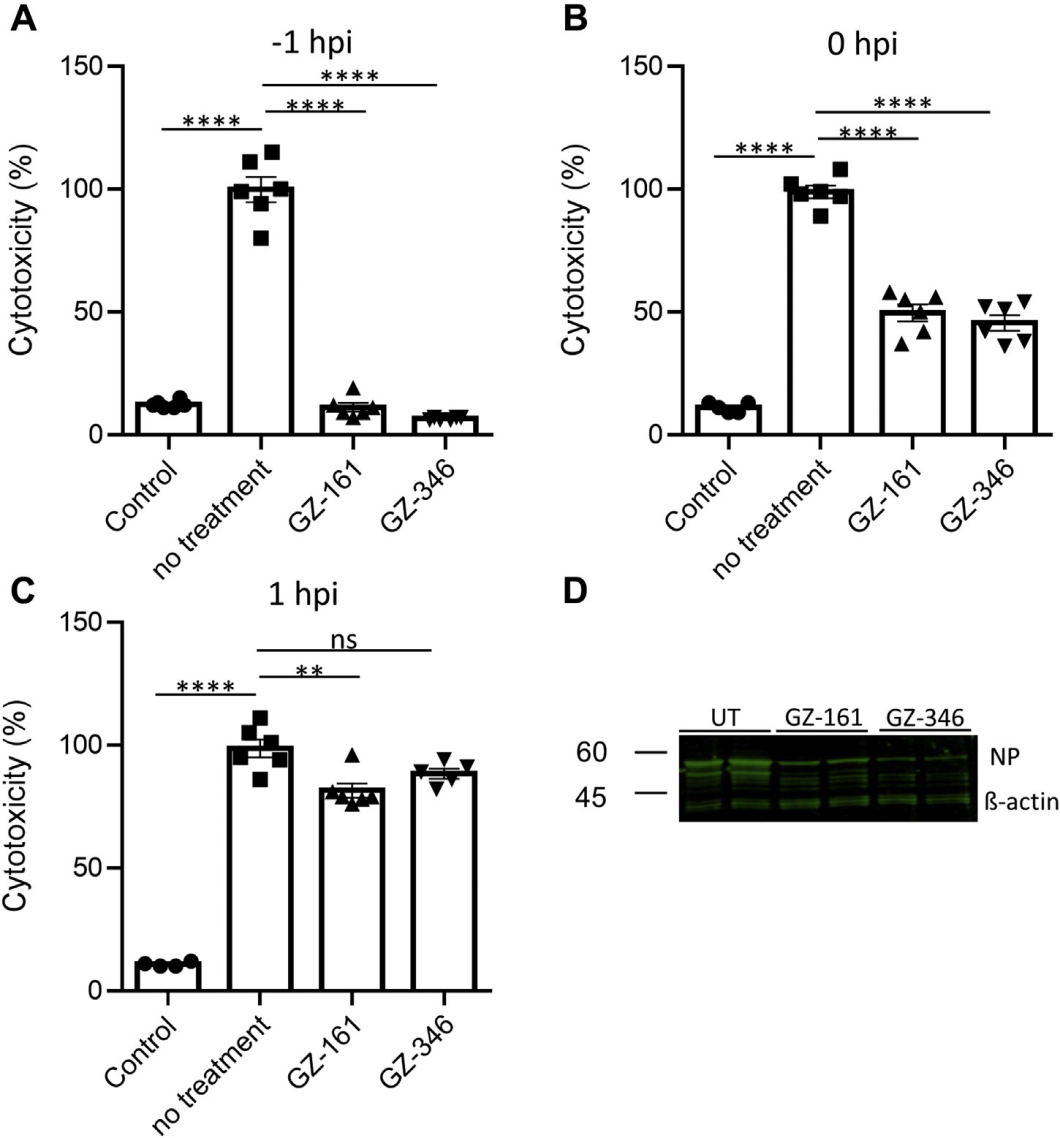
**GCS inhibitors disrupt early stages of SARS-CoV-2 replication.***A*–*C*, Vero E6 cells were infected with SARS-CoV-2 at a multiplicity of infection (MOI) of 0.01 in the presence of GZ-161 (10 μM) or GZ-346 (10 μM). GCS inhibitors were added to the media 1 h before infection (−1 h after infection, *A*), immediately after virus absorption (0 h after infection, *B*) and 1 h after infection (1 h after infection, *C*). Forty eight hours after infection, cell death was measured by CytoTox-Fluor Cytotoxicity Assay. The percentage of dead cells was calculated compared with infected untreated cells. Data are the means of 4 to 6 replicates ±SEM. Statistical analysis was performed using the one-way ANOVA test followed by Tukey's multiple comparison tests. ∗∗∗∗*p* < 0.0001. ∗∗*p* < 0.01. *D*, reduced levels of SARS-CoV-2 nucleocapsid protein (NP) in GCS inhibitor–treated cells 5 h after infection. Vero E6 cells were infected with SARS-CoV-2 at a multiplicity of infection (MOI) of 5 in the presence of GZ-161 (10 μM), GZ-346 (10 μM), or untreated (UT). GCS inhibitors were added to the media 1 h before infection. The expression of β-actin protein was used as a loading control. Results are representative of triplicates. GCS, glucosylceramide synthase; ns, not significant; SARS-CoV-2, severe acute respiratory syndrome coronavirus 2.

Next, the ability of GCS inhibitors to reduce the cytopathic effect (CPE) of SARS-CoV-2–infected cells was examined. Vero E6 cells were incubated with 10-μM GZ-161, or GZ-346, 1 h before infection with SARS-CoV-2, and cell cytotoxicity was measured at 48 h after infection. Treatment with GZ-161 or with GZ-346, 1 h before infection, completely eliminated SARS-CoV-2–induced cell cytotoxicity (Fig. 4*A*). Taken together, these results demonstrate that GZ-161 and GZ-346 have an antiviral effect on the SARS-CoV-2 clinical isolate *in vitro*, with a single dose able to significantly inhibit viral replication within 24 to 48 h.

### GCS inhibitors disrupt early stages of SARS-CoV-2 replication

To determine which stage of SARS-CoV-2 infection cycle was affected by GCS inhibitors, a time-of-addition assay was performed. As shown in Figure 4, *A*–*C*, inhibition was most effective when GCS inhibitors were added 1 h before infection and was not effective when given 1 h after infection (Fig. 4*C*). When GCS inhibitors were added immediately after SARS-CoV-2-attachment (0 h after infection), viral replication was significantly reduced by ∼50% (Fig. 4*B*). The time dependence of the inhibitory effect of the compound suggests that its anti-SARS-CoV-2 activity may be due to inhibition of early steps in the SARS-CoV-2 replication cycle. To further examine the stage which is being interrupted by GCS inhibitors in the life cycle of SARS-CoV-2, Vero E6 were infected with high MOI of 5 to ensure single-cycle infection. Cells were incubated with 10-μM GZ-161 or GZ-346 1 h before infection with SARS-CoV-2. Five hours after infection, SARS-CoV-2 nucleocapsid (N) levels were determined (Fig. 4*D*). GZ-161 and GZ-346 significantly reduced the levels of N protein in infected cells. Thus, our data suggest that GCS inhibitors inhibit SARS-CoV-2 infection cycle after attachment of the virus and before the translation of subgenomic proteins.

### Inhibition of influenza virus A/PR/8/34 (H1N1) by GCS inhibitors

**Figure 5:**
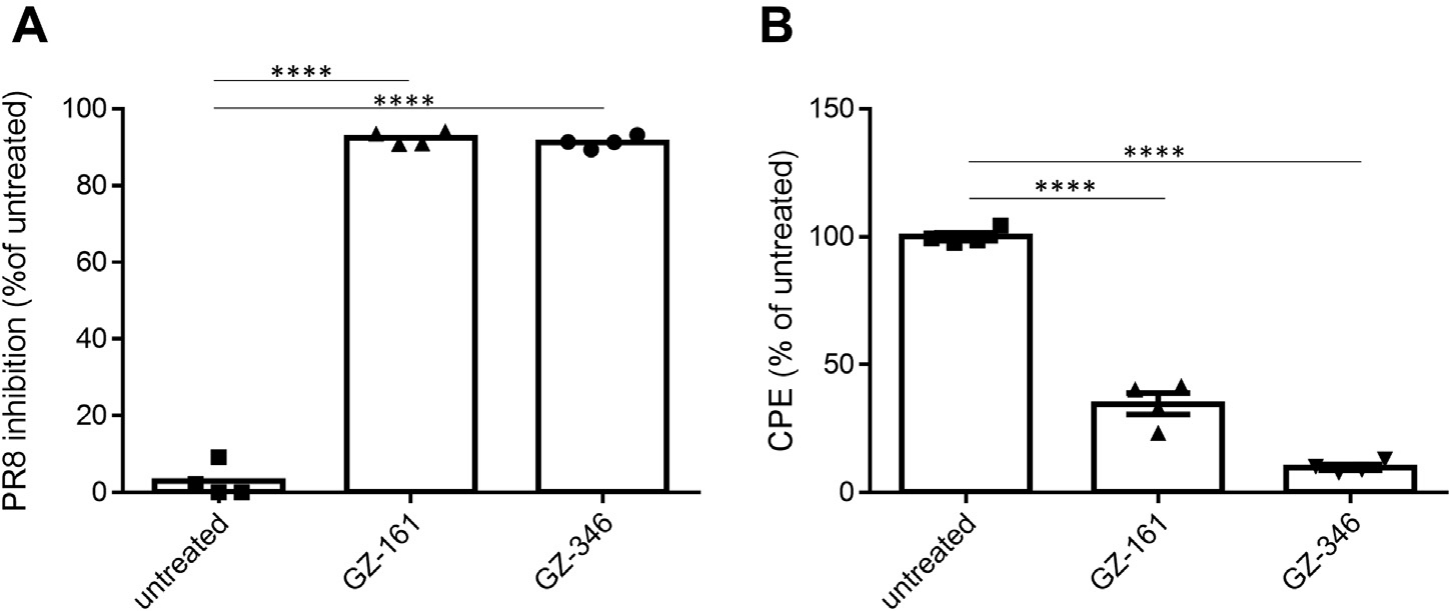
**Inhibition of influenza virus A/PR/8/34 (H1N1) by glucosylceramide synthase inhibitors.** MDCK cells were treated with GZ-161 or GZ-346 (10 μM). One hour later, cells were infected with influenza virus A/PR/8/34 (H1N1) diluted in Eagle’s minimal essential medium containing 2 μg/ml trypsin (MOI, 0.1). *A*, a bar graph showing the effect of GZ-161 and GZ-346 on viral release. Viral release to the media was measured by real-time PCR 8 h after infection and the percentage of inhibition was calculated. Data are the means of four replicates ±SEM. *B*, GCS inhibitors reduce the cytopathic effect of PR8. Cell death was measured 24 h after infection by the LDH cytotoxicity assay kit. The percentage of cytotoxicity was calculated. Data are the means of four replicates ±SEM. ∗∗∗∗*p* < 0.0001 *versus* infected untreated. CPE, cytopathic effect; GCS, glucosylceramide synthase; LDH, lactate dehydrogenase; MDCK, Madin–Darby Canine Kidney; MOI, multiplicity of infection; PR8, influenza virus A/PR/8/34 (H1N1).

It was recently shown that *Ugcg* KO cells demonstrate a reduction in the replication of the respiratory RNA virus Influenza A virus, a member of the family Orthomyxoviridae (21). Thus, we further examined the antiviral activity of GCS inhibitors against Influenza A virus. Madin–Darby Canine Kidney (MDCK) cells were incubated with 10-μM GZ-161 or GZ-346 1 h before infection with mouse-adapted influenza virus A/PR/8/34 (H1N1) (PR8). The supernatant was harvested 8 h after infection and analyzed by qPCR for the detection of viral RNA in the culture media (Fig. 5*A*). Approximately 90% reduction in viral RNA was measured in samples treated with GZ-161 or GZ-346 compared with the vehicle DMSO (untreated). In addition to their ability to inhibit PR8 replication, the ability of GCS inhibitors to reduce the CPE of PR8-infected cells was further examined. MDCK cells were incubated with 10-μM GZ-161 or GZ-346 1 h before infection with PR8 (MOI, 0.1) and lactate dehydrogenase release to the supernatant was measured at 24 h after infection as an indication for cell disintegration. Both GZ-161 and GZ-346 significantly reduced PR8-induced cytotoxicity by 65% and 90%, respectively (Fig. 5*B*).

## Discussion

In this study, we demonstrate that the GCS inhibitors GZ-161 and GZ-346 inhibit viral replication of SARS-CoV-2. The importance of GSL biosynthesis in the viral life cycle was demonstrated recently for Influenza virus and severe fever with thrombocytopenia syndrome virus (21, 22). Moreover, iminosugars are known for their broad-spectrum antiviral activity, presumably due to their mechanism of action as endoplasmic reticulum–resident α-glucosidases I and II inhibitors (23). 1-Deoxynojirimycin (DNJ) iminosugar derivatives inhibit *in vitro* production of infectious viruses including DENV (24, 25), hepatitis B virus (26, 27), hepatitis C virus (28), HIV (29, 30), and influenza A virus (31). Antiviral efficacy of the iminosugar N-butyl–DNJ (NB-DNJ, miglustat, ZAVESCA) has been further demonstrated *in vivo* against DENV infection (32). Although these reports present strong circumstantial evidence that inhibition of endoplasmic reticulum α-glucosidase activity is the cause of iminosugar antiviral activity (33), the ubiquity of d-glucose in metabolism suggests that other pathways may be equally affected by iminosugar treatment. Indeed, NB-DNJ has been approved for clinical use since 2002 as a second-line treatment for Gaucher disease (34)—an LSD. In this context, NB-DNJ is used as an inhibitor of GCS, to reduce production of GSLs that accumulate due to a deficiency in GlcCer degradation (35). Thus, the broad antiviral activity of NB-DNJ can also be explained by its inhibition of GCS, suggesting the GSL synthetic pathways may be therapeutic targets for a broad range of viral infection. Unlike the iminosugar *N*B-DNJ, eliglustat is a ceramide analogue that inhibits UDP-GCS without inhibiting of intestinal glycosidases (lactase, maltase, sucrase), α-glucosidase I and II, as was evaluated in *in vitro* cell-based and cell-free assays (36, 37).

The mechanism by which GCS inhibitors block viral replication is not fully resolved. SLs play a significant role in endocytosis and thus may play a major role in virus penetration to the cell. Previous works showed that knocking out *Ugcg* impaired the entry of Influenza virus and thrombocytopenia syndrome virus by endocytosis (21, 22). This is consistent with our data showing interruption of early stages of SARS-CoV-2 replication. Whether the same mechanism of inhibition is relevant to SARS-CoV-2 needs to be elucidated. Moreover, whether the antiviral effect of GCS inhibitors is due to decreased levels of GlcCer and/or other GSLs or due to elevated levels of ceramide needs to be further explored.

The antiviral effect of GCS inhibitors on viruses of three different families (*i.e.*, Bunyaviridae, Orthomyxoviridae, and Coronaviridae) suggests a key role of the GSL synthesis pathway in viral infection. One advantage of targeting host proteins used by multiple viruses over targeting specific viral proteins is it being less prone to the development of resistance to the drug through mutations. Although side effects may be of particular concern for such treatments, another advantage of targeting host protein is the availability of many approved drugs against host proteins, allowing for drug repurposing. The main advantage of repurposing approved drugs is that they have already proven to be sufficiently safe, they have successfully passed clinical trials and regulatory scrutiny, and they have already undergone postmarketing surveillance (38). Although the inhibitor NB-DNJ affects multiple host targets, specific inhibition of GCS is now possible using GCS inhibitors that are currently available. Eliglustat is an oral therapy approved in the European Union (2015) and the United States (2014) as a first-line treatment for adults with type 1 Gaucher disease who have compatible CYP2D6 metabolism phenotypes. Phase I studies in healthy volunteers revealed limited toxicity with an excellent pharmacodynamic response (37). A phase 3 trial in which patients received eliglustat daily (50 or 100 mg twice daily) for 9 months showed mild or moderate adverse events (39, 40).

We have shown that commercially available, United States Food and Drug Administration-approved analogues, pharmacological inhibitors specific for GCS have an antiviral activity against both SARS-CoV-2 and Influenza virus. We suggest that examining the effects of these drugs *in vivo*, as well as against other viruses, can be beneficial and yield novel treatments for viral infection.

## Experimental procedures

### Cells

Vero E6 (ATCC CRL-1586) and MDCK cells (ATCC CCL-34) were kindly provided by Michal Mandelboim (Tel Aviv University, Israel). Cells were maintained in Dulbecco’s modified Eagle’s medium supplemented with 10% heat-inactivated fetal calf serum, nonessential amino acids, 2-mM L-glutamine, 100 units/ml penicillin, 100 μg/ml streptomycin, and 1.25 units/ml nystatin at 37 °C under a 5% CO_2_/95% air atmosphere.

### Viruses

SARS-CoV-2 (GISAID accession EPI_ISL_406862) was kindly provided by Bundeswehr Institute of Microbiology, Munich, Germany. Stocks were prepared by infection of Vero E6 cells for 2 days, a time point in which CPE becomes visible. Media were collected and clarified by centrifugation before being aliquoted for storage at −80 °C. Titer of the stock was determined by plaque assay on Vero E6 cell monolayers. Influenza virus A/Puerto Rico/8/34 H1N1 (PR8) was kindly provided by Michal Mandelboim (Tel Aviv University, Israel).

### GCS inhibitors

The compounds GZ-161 ((S)-quinuclidin-3-yl (2-(2-(4-fluorophenyl)thiazol-4-yl)propan-2-yl)carbamate) and GZ-346 ((1R,2R)-nonanoic acid[2-(2′,3′-dihydro-benzo [1,4] dioxin-6′-yl)-2-hydroxy-1-pyrrolidin-1-ylmethyl-ethyl]-amide-l-tartaric acid salt) were obtained from Sanofi. The compounds were stored as 20-mM and 5-mM stock solutions in DMSO or in PBS, respectively, at −20 °C until use.

### Quantitative real-time PCR

The supernatant was collected, centrifuged in a tabletop centrifuge for 5 min at maximum speed and stored at −80 °C. RNA was extracted by using the Qiagen viral RNA extraction kit as per the manufacturer’s instructions. RNA load in the media was determined by quantitative real-time PCR. Real-time PCR was conducted with SensiFAST Probe Lo-ROX One-Step Kit (Bioline, 78005) and analyzed with the 7500 Real-Time PCR System (Applied Biosystems). The PFU equivalent per ml was calculated from the standard curve generated from virus stocks. Quantitative PCR primers and probes for the detection of SARS-CoV-2 N1 were obtained from IDT (2019-nCoV CDC EUA Kit, cat #10006606). Quantitative PCR primers and probes for the detection of PR8: PR8-PA-FW: CGGTCCAAATTCCTGCTGA; PR8-PA-RW:CATTGGGTTCCTTCCATCCA; PR8-PA-Probe: CCAAGTCATGAAGGAGAGGGAATACCGCT.

### Inhibition of SARS-CoV-2 virus in the cell culture

Vero E6 cells were seeded at a density of 1.5 × 10^5^ cells per well in 24-well plates. After overnight incubation, cells were treated in 4 replicates with GZ-161 or GZ-346. Cells were infected 1 h later with SARS-CoV-2 (MOI, 0.01 or 5, as indicated). The supernatant was collected 24 h after infection or 10 h after infection for quantitative PCR and for PFU quantification. For PFU quantification, Vero E6 cells were seeded at a density of 4 × 10^5^ cells per well in 12-well plates. After overnight incubation, cell monolayers were infected with serial dilution of media and 30 to 35 PFU/well of live virus served as control and incubated for 48 h at 37 °C. Then, the inhibitory capacity of GCS inhibitors was assessed by determining the numbers of plaques compared with untreated cells. Cell viability was determined 48 h after infection by using the Cell Proliferation Kit (XTT based) (Biological Industries, 20-300-1000) according to manufacturer’s protocol. The percentage of inhibition was calculated by subtracting the ratio of the PFU between treated and untreated cells from 1. All experiments involving SARS-CoV-2 were conducted in a BSL3 facility in accordance with the Israel Institute for Biological Research regulation.

### *In vitro* DsiRNA transfection assay

Vero E6 cells were seeded at a density of 1.5 × 10^5^ cells/well in a 24-well plate and allowed to attach overnight in the growth medium. For DsiRNA knockdown of *Ugcg*, cells were transfected with 20 nM of nontargeted or *Ugcg*-specific DsiRNA (IDT) using Lipofectamine RNAiMAX reagent (Invitrogen) at 37 °C. The transfection was performed according to the manufacturer’s instructions using 1.5-μl Lipofectamine RNAiMAX in a total volume of 500 μl growth medium without antibiotics. Cells were allowed to grow for 1 day at 37 °C, 5% CO_2_, and were then analyzed for *Ugcg* mRNA expression or infected with SARS-CoV-2. Mock-transfected cells were used as control.

### Cell cytotoxicity

Cell death was determined by CytoTox-Fluor Cytotoxicity Assay (Promega, G9262) according to manufacturer’s protocol.

### Western blotting

Cells were washed twice with PBS and lysed in RIPA buffer (Merck, R0278) supplemented with a protease inhibitor cocktail (Merck, P8340). Samples were sonicated twice for 5 s to fragment DNA and boiled for 5 min to denature proteins. Lysates were resolved on 12% SDS-PAGE (Genscript, ExpressPlus PAGE Gel, M01212) and subsequently transferred onto a nitrocellulose membrane (Thermo Fisher Scientific, iBlot 2 Transfer Stacks, nitrocellulose, IB23002). The membrane was blocked with 5% bovine serum albumin (Biological industries, 03-010-1B) in PBS-0.05% Tween 20 and incubated overnight with a primary antibody at 4 °C. After washing, the membrane was incubated with IRDye 800CW goat anti-rabbit secondary antibody (LI-COR, 926-32211) for 1 h at 20 °C. The membrane was washed with PBS-0.05% Tween and developed using Odyssey-CLx imaging system (LI-COR). Primary antibodies include rabbit anti-β-actin (Abcam, AB8227) and rabbit anti-SARS-CoV-2 nucleocapsid (1:2000, kindly provided by Amir Ben-Shmuel, Israel Institute for Biological Research, Israel).

### Inhibition of influenza virus in the cell culture

MDCK cells were seeded at a density of 5 × 10^5^ cells per well in 6-well plates. After overnight incubation, cells were treated in four replicates with GZ-161 or GZ-346. One hour later, cells were infected in a serum-free medium containing 0.5 μg/ml TPCK-trypsin with PR8 at MOI 0.1. Supernatants were collected 8 h after infection for qPCR. Cell cytotoxicity was determined 24 h after infection by LDH Assay (Cytotoxicity) (ab65393) according to manufacturer’s protocol. The percentage of inhibition was calculated by subtracting the ratio of PFU between treated and untreated cells from 1.

### Statistical analysis

Statistical analyses were performed with a two-tailed unpaired *t* test or one-way ANOVA test followed by Tukey's multiple comparison tests, as indicated in the legends. *p* values are indicated by asterisks in the figures as follows: ∗*p* < 0.05, ∗∗*p* < 0.01, ∗∗∗*p* < 0.001, and ∗∗∗∗*p* < 0.0001. Differences with a *p* value of 0.05 or less were considered significant. The exact value of n is indicted in the figure legends. Data for all measurements are expressed as the means ± SEM. Analyses were performed using GraphPad Prism software, version 6.0.

## Data availability

All data required for the conclusions made here are contained within the article. Any other data are available at request from the authors. Data are in United States Provisional Patent Application No. 63/014386 “Glucosylceramide synthase inhibitors for prevention and treatment of viral diseases”.

## Conflict of interest

The authors declare that they have no conflicts of interest with the contents of this article.
